# Positive sparse coding of natural images: a theory for simple cell tuning

**DOI:** 10.1186/1471-2202-14-S1-P250

**Published:** 2013-07-08

**Authors:** David GT Barrett, Sophie Denève, Christian K Machens

**Affiliations:** 1 Institut D'Étude de la Cognition, École Normale Supérieure, Paris, France; 2 Champalimaud Centre for the Unknown, Lisbon, Portugal

## 

One of the most celebrated results in neuroscience is the observation that simple cells in the visual cortex are tuned to the orientation and polarity of edges in visual stimuli [1]. While orientation tuning has been the subject of intense investigation, the polarity tuning of cells is poorly understood; a simple cell responds either to a bright edge with dark flanks, or to the opposite polarity, a dark edge with bright flanks. We ask, what is the function of this polarity tuning, if any, and how does it arise from the underlying neural circuitry?

Perhaps the most influential functional theory of simple cell tuning is sparse coding [2]. According to this theory, the function of simple cells is to represent natural images sparsely (using as few active neurons as possible). This representation is desirable because it is metabolically and computational efficient. It explains orientation tuning because sparse coding neurons are tuned to edge-like stimuli at different orientations. However, there are two problems with this 'classical' sparse coding (Table 1). First of all, it predicts that every cell is tuned to both polarities, whereas simple cells only respond to a single polarity. Secondly, it requires neurons to represent images using negative firing rate values, which is clearly impossible for a spiking neural network.

**Table 1:** Tuning properties

	**Classical sparse coding**	**Positive** **sparse coding**	**Simple cells**
Orientation tuning	**✓**	**✓**	**✓**
Position tuning	**✓**	**✓**	**✓**
Polarity tuning	**✗**	**✓**	**✓**
Positive firing	**✗**	**✓**	**✓**

**Figure 1:**
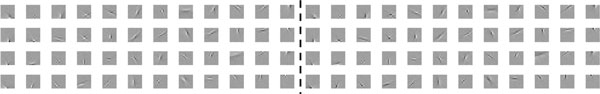
**Positive sparse coding filters of natural images**. Each image is the sparse coding filter for a single neuron. These are tuned to orientation and polarity (left side neurons tuned to the opposite polarity of right side neurons).

We solve both of these problems, simultaneously, by proposing that simple cells provide a positive sparse coding of natural images. This is similar to 'classical' sparse coding but with positive firing rates alone. We find that positive sparse coding neurons have both orientation tuning and polarity tuning (Figure 1, Table 1). Orientation tuning emerges because natural images typically contain edges at many different orientations, and a sparse code will capture these natural statistics. Polarity tuning emerges as a natural consequence of the positivity constraint because a neuron with positive firing rates cannot represent edges with both polarities on its own.

This functional theory leads naturally to a spiking neural network implementation for simple cells, because spiking network models necessarily have positive firing rates. We find that a network of tightly balanced leaky integrate-and-fire neurons can produce spike trains that optimise our positive sparse coding cost function. In this way, we can marry spiking constraints with neural function to understand the orientation and polarity tuning of simple cells.
